# Rare Presentations of Cytomegalovirus Infection in Renal Allograft Recipients

**DOI:** 10.5812/numonthly.1844

**Published:** 2012-03-01

**Authors:** Mohammadreza Ardalan

**Affiliations:** 1 Department of Nephrology, Tabriz University of Medical Sciences, Tabriz, IR Iran; 2 Mario Negri Institute of Pharmacological Research, Bergamo, Italy

**Keywords:** Cytomegalovirus, Lymphohistiocytosis, Hemophagocytic, Kidney Transplantation

## Abstract

Cytomegalovirus is the most common viral infection after kidney transplantation. Clinical presentations of cytomegalovirus infection range from asymptomatic infection to organ-specific involvement. Most symptomatic infections manifest as fever and cytopenia. The gastrointestinal tract is the most common site of tissue-invasive infection, often presenting as diarrhea or gastrointestinal bleeding. Gastrointestinal obstruction, perforation, thrombosis of large gastrointestinal veins, splenic artery thrombosis, and pancreatitis are rare gastrointestinal presentations of cytomegalovirus infection. Renal-allograft ureteral stricture and skin involvement are other rare presentations of cytomegalovirus infection. hemophagocytic syndrome, thrombotic microangiopathy, adrenal insufficiency, and renal allograft artery stenosis are other rare symptoms of cytomegalovirus infection.

## 1. Introduction

Cytomegalovirus (CMV) is the most common viral infection contracted after kidney transplantation, and its clinical presentation ranges from asymptomatic infection to organ-specific involvement (1). Most symptomatic infections manifest as fever, fatigue, and cytopenia. The gastrointestinal tract is the most common site of tissue-invasive CMV infection, with abdominal pain and diarrhea as the most prominent symptoms. In severe cases, gastrointestinal hemorrhage and perforation occur. CMV infection can involve the liver, lungs, heart, pancreas, and kidneys. Chorioretinitis, which is a common manifestation in HIV-infected patients, is very rare in solid-organ recipients (1-3). Through its ability to modulate the immune system, CMV infection increases the risk of other opportunistic infections, including human herpes viruses (HHV-6 and HHV-7), Epstein-Barr virus, nocardia, mycobacteria, and fungi such as Aspergillus and candida (1, 4, 5). Patients with CMV infection are more likely to experience acute and chronic rejection. The greatest CMV-induced damage tends to occur within the transplanted organ itself; that is, bronchitis obliterant and pneumonitis in lung-transplant recipients, hepatitis in liver-transplant recipients, and transplant vasculopathy in heart-transplant recipients. CMV is also considered a risk for the development of malignancy and posttransplant diabetes (1-3). Active CMV infection can be confirmed by specific antibody assays, detection of inclusion bodies within the infected cells, antigen staining by immunohistochemical methods, detection of viremia by polymerase chain reaction (PCR), and CMV-DNA in peripheral blood leukocytes (1, 3, 6).

## 2. Vascular Involvement 

CMV-induced vasculopathy and thrombosis have been reported in both immune-compromised and immune-competent individuals. Endothelium is a latency site for the CMV virus. Thrombosis of the jugular, cerebral, retinal, and upper-extremities veins have been reported as a complication of CMV infection in HIV-infected patients (7). Some cases of arterial and venous thrombosis have been repoted among immune-competent individuals (8-10). CMV infection plays an important role in atherosclerosis of the coronary arteries after heart transplantation and in restenosis after angioplasty (8, 9). CMV infection especially affects the gastrointestinal microvasculature and manifests as colonic ulceration, bleeding, and perforation. Enlarging of the colonic vein and splenic venous and arterial thrombosis have been associated with CMV infection (10). CMV infection induces endothelial inflammation, increasing leukocyte and platelet adhesion and endothelial expression of Vwf, E-selectin, and vascular cell adhesion molecule-1 [VCAM-1 (11, 12]). The plasma levels of factor VIII, fibrinogen, protein S, platelet-derived growth factor, and transforming growth factor–B are higher in patients with active cytomegalovirus infection (13). The presence of antiphospholipid antibodies increases the risk of CMV-induced vascular damage. Extensive splenic infarct has been reported after CMV infection in a patient with heterozygous mutation of factor V leiden (14). one of the gene products of CMV, IE84, inhibits P53-mediated apoptosis and enhances vascular smooth-muscle-cell proliferation (15). It has been shown that CMV is a cause of aortic inflammation in mice infected with CMV (16). In a case study of induced CMV, acute transplant renal artery stenosis was resolved after ganciclovir therapy without any need for angioplasty (17). CMV infection has been suggested in the pathogenesis of polyarteritis nodosa (PAN), systemic necrotizing vasculitis, and Kawasaki disease (16, 18, 19). It has been reported that carotid artery intima thickening disappears after appropriate therapy for acute CMV infection (20).

## 3. Urologic Complications 

In two early reports, CMV infection was proposed as a cause of renal allograft ureteral stricture (21, 22). later reports confirmed this idea. A review of reported cases showed that induction therapy with antilymphocyte globulin and antirejection therapy with antilymphocyte globulin and intravenous methylprednisolone are the most prominent risk factors in the development of CMV-induced urologic complications. Symptoms often begin around 4 to 5 weeks after an intensive immunosuppressive therapy. Patients do not show major symptoms of viremia and often present with low-grade fever, supra-pubic pain, and rising serum creatinine. In one report, CMV cystitis began 10 days after antirejection therapy (23). Ultrasounds are useful for diagnosing ureteral stricture. Serologic tests often show an active CMV infection. Ureteral stricture is often severe and found in the upper or middle ureter or in the ureteropelvic junction. At the time of this writing, all CMV studies using histologic examinations have shown extensive inflammation, ureteral necrosis, and intranuclear inclusion bodies in endothelial and vascular smooth muscle cells. All patients in previous studies were treated with surgical resection and reanastomosis (Table 1; [24-27]).

**Table 1:** Ureteral Stenosis in CMV Infection

**Age/Sex**	**CMVR a/D a**	**Induction**	**Rejection**	**Signs/Time b**	**Diagnosis/Treatment**
42/F a	+/+	ATG a /5d	-	Suprapubic pain, hydronephrosis /1 mo	PP65 antigenemia & histology c/resection & re-anastomosis (24)
50/M a	+/-	ATG a /5d	-	Fever, suprapubic pain, leukopenia, hydronephrosis /25 d	Histology /resection & re-anastomosis (24)
45/M	IgG+/IgG-	Basiliximab	41 d, MTP a	Allograft dysfunction & hydronephrosis/ 65 d	Histology/resection & re-anastomosis (25)
31/F	-/+, (No prophylaxy)	-	2 wk, MTP	Hydronephrosis & oliguria/2 mo	Histology/resection & re-anastomosis (26)
26/F	+/-	-	8 d, MTP	Suprapubic pain & cystitis /18 d Hydronephrosis /45 d	Histology/ resection & re-anastomosis (23)
62/F	+/-	-	8 d, MTP	Fever, suprapubic pain, hydronephrosis/ 6 wk	Histology/resection & re-anastomosis
35/M	+/-	-	10 d, MTP	Fever, suprapubic pain Hydronephrosis	Histology/resection & re-anastomosis (23)
56/M	+/-	-	15 d, MTP	Allograft dysfunction & hydronephrosis /8 wk	Histology/resection & re-anastomosis (23)
62/M	+/-	-	10 d, MTP	Lower extremity edema, Periallograft urine Collection	Histology/resection & re-anastomosis (27)

^a^Abbreviations: ATG, antithymocyte globuline; CMV, cytomegalovirus; D, donor; F, female; M, male; MTP, methyl prednisolone; R, recipient.

^b^Time; time of occurrence of the complication after transplantation.

^c^Histology; specific pathologic changes including CMV inclusion bodies and owl’s eye lesion found in a histological study of involved organs and detection of CMV antigens by immuno-histochemical staining.

## 4. Gastrointestinal Complications 

Symptomatic CMV disease of the gastrointestinal tract occurs in about 5–10% of renal-transplant recipients. Gastrointestinal involvement can be localized or extensive. Ulcers of the esophagus, stomach, small intestine, and colon also manifest as dysphagia, abdominal pain, diarrhea, bleeding, or perforation. hepatitis can occur, especially after a liver transplant. CMV vasculitis can affect the gastrointestinal tract significantly, causing colonic ulceration, bleeding, and perforation. Colonic complications are usually severe and vascular in origin. A study using a pathologic examination revealed ulcerations and necrosis with scattered CMV inclusions (28). Duodenal vasculitis, intermittent small-bowel obstruction, acute pancreatitis, bloody ascites, colonic obstruction, and painful hemorrhoids have been reported as rare gastrointestinal presentations of CMV infection (28-36). Rare gastrointestinal presentations of CMV infection in kidney-transplant recipients are shown in Table 2.

**Table 2:** Rare Gastrointestinal Manifestations of CMV Infection

**Age, y**	**Sex**	**Recipient**	**Donor**	**Time** **b**	**Type of transplantation**	**Induction or antirejection** **medication**	**Signs**	**Diagnosis**	**Treatment**
37	F a	+	+	30 d	kidney	Anti-CD52	Abdominal pain, bloody peritoneal fluid, leukopenia	Peritoneal fluid RT-PCR c, CMV-pp65 d	Peritoneal-catheter removal, medical therapy (34)
41	F	-	-	6 mo	-	ATG a	Abdominal pain, Pancreatitis	RT-PCR	Medical therapy (36)
46	F	+	+	2 mo	kidney	Basiliximab	Fever, abdominal pain, duodenal vasculitis	RT-PCR	Histology e, medical therapy (33)
50	M a	+	+	8 wk	Kidney, Liver	ATG	Abdominal pain, duodenal vasculitis, obstruction	RT-PCR	Histology, surgical resection (35)
58	F	+	+	6 mo	kidney	-	Fever, leukopenia, thrombocytopenia, painful hemorrhoid	CMV-PP65 Ag	Histology (32)
44	M	?	-	2 mo	Kidney	OKT3 F	Abdominal pain, diarrhea, leukopenia, large colonic veins thrombosis	Histology	Total colectomy (30)
62	M	+	+	4 y	heart	-	Abdominal pain, hematochezia, colonic stenosis	Histology	Surgical resection (31)

^a^Abbreviation: ATG, antithymocyte globuline; F, female; M,male

^b^Time at which the complication presents after transplantation.

^c^RT-PCR, detection of CMV-DNA in plasma or other bodily fluid by real-time polymerase chain reaction.

^d^CMV-pp65, detection of CMV-PP65 antigen.

^e^Histology, that pathologic changes including CMV inclusion bodies have been found in involved tissues.

^F^OKT3 is an anti Tcell murine monoclonal anibody.

## 5. Cytomegalovirus Skin Involvement 

Skin involvement is extremely rare in CMV infections. It should be considered when multiple anogenital, axillary, scrotal, and penile ulcers appear in an immune-compromised individual and are not responding to conventional antibiotic therapy (37). Painful ulceration of the tongue was reported as one manifestation of CMV infection in two renal-transplant patients. A combination of multiple anogenital ulcerations and generalized maculopapular rashes has been reported as a rare symptom of CMV infection (38). In one study, CMV vasculitis was the cause of perineal lesion in patients with partial response to anti hSV therapy. Perineal nerves are a potential site of CMV reactivation (39). Most cytopathic changes occur in the dermis, particularly within the vascular endothelium. CMV has been considered in the pathogenesis of immune-based cutaneous lesions such as cutaneous vasculitis and scleroderma (40).

The dermis is a relatively inhospitable site for CMV replication in that skin involvement occurs only in severely immune-compromised individuals (41). A skin biopsy can identify the disease before cultural or serologic conurbation. Early diagnosis and specific treatment can reduce the mortality rate for this potentially fatal condition (Table 3).

**Table 3:** Coetaneous Manifestation of CMV Infection

**Age/Sex**	**Time b**	**Presentation**	**Diagnosis/Treatment**
56/M a	4 mo	Macula-papular skin lesions colonic ulcer	Histology c/medical therapy (37)
31/M	-	Multiple penile ulcers	Histology/medical therapy (38)
62/F a	3 mo	Multiple perineal ulcer	Histology/medical therapy (41)
62/F	3 mo	Painful tongue ulcer, diffuse petechial Lesion on face. Diarrhea, low fever	Histology, CMV-PP65 d RT-PCR e, ulcer swabbing medical therapy (41)
39/F	7 y	peri-anal ulcer	Histology/surgical resection, medical therapy
34/M	6 mo	Fever, tongue ulcer, pinnal nodule, non productive cough, neutropenia	Histology (pinnal nodule) /medical therapy (41)
52/M	2.5 mo	Fever, vesicular ulcer on axilla, scrotum and penis	Histology, RT-PCR/medical therapy

^a^Abbreviations: F, female; M, male

^b^Time, time of presentation after transplantation

^c^Histology, specific pathologic changes including CMV inclusion bodies, owl,s eye lesion, and detection of CMV antigens by immuno-histochemical staining

^d^CMV-pp65, detection of CMV-PP65 antigen

^e^RT-PCR, detection of CMV-DNA in plasma or other bodily fluid by real-time polymerase chain reaction

## 6. CMV and Malignancy 

A possible relationship between CMV infection and cancer has been suggested in several studies. In addition to epidemiologic evidence, the detection of CMV-DNA, mRNA, or antigens in tumor tissues in some studies suggests a possible role of CMV infection in the pathogenesis of several human malignancies (42-45). human CMV nucleic acids and proteins have been discovered in a high percentage of low- and high-grade malignant gliomas (45). CMV is found within the breast epithelial cells, which suggests that it may play a role in the neoplastic process (46). US28 is a costimulatory chemokine receptor encoded by CMV. Transgenic coexpression of the US28 ligand, CCl2, which is an inflammatory chemokine, increases intestinal endothelial cell proliferation and the development of intestinal neoplasia (47).

## 7. Miscellaneous Presentations

Hemophagocytic syndrome, also referred to as macrophage activation syndrome, is a rare, systemic proliferation of benign monocyte–macrophage lineage (48-50). hemophagocytic syndrome has been reported as a rare complication of CMV infection in renal-transplant recipients. Patients often present with fever, splenomegaly anemia, pancytopenia, and elevated aminotransferase levels. Bone marrow examination often discloses extensive hemophagocytosis (48, 49, 51, 52).

After kidney transplantation, thrombotic microangiopathy (TMA) can reoccur. CMV infection can be a trigger for TMA in kidney-transplant recipients, and TMA can occur shortly after a transplant or even years later (53-55). A case of bilateral CMV retinitis presented as a floater was reported in a case study of a woman with systemic lupus erythematous who was undergoing hemodialysis. the condition improved after Ganciclovir therapy (56). In another case study, acute unilateral sensorineural hearing loss was reported as a sign of CMV infection 8 months after simultaneous pancreas and kidney transplant. The recipient was CMV-antibody negative and the donor was CMV-antibody positive, and CMV developed 2 months after completing 6 months of Valganciclovir prophylactic therapy. Hearing loss improved 5 weeks after intravenous ganciclovir therapy (57). Concomitant CMV pneumonitis and subacute thyroiditis were reported in a case study of a recipient of a bone-marrow transplant. The condition successfully responded to treatment with ganciclovir and prednisolone (58). CMV-induced adrenal insufficiency is a known condition in HIV-infected patients and has also been reported in one renal-transplant recipient, whose condition improved after increasing his corticosteroid dosage (59).

Cytomegalovirus is a common viral infection after kidney transplantation. Familiarity with unusual manifestations of CMV infection can help the clinician to better care and manage treatment for renal-transplant recipients.
